# A Toolbox of Criteria for Distinguishing Cajal–Retzius Cells from Other Neuronal Types in the Postnatal Mouse Hippocampus

**DOI:** 10.1523/ENEURO.0516-19.2019

**Published:** 2020-01-22

**Authors:** Max Anstötz, Gianmaria Maccaferri

**Affiliations:** Department of Physiology, Feinberg School of Medicine, Northwestern University, Chicago, IL 60611

**Keywords:** development, GABA, interneuron, network, p73, reelin

## Abstract

The study of brain circuits depends on a clear understanding of the role played by different neuronal populations. Therefore, the unambiguous identification of different cell types is essential for the correct interpretation of experimental data. Here, we emphasize to the broader neuroscience community the importance of recognizing the persistent presence of Cajal–Retzius cells in the molecular layers of the postnatal hippocampus, and then we suggest a variety of criteria for distinguishing Cajal–Retzius cells from other neurons of the hippocampal molecular layers, such as GABAergic interneurons and semilunar granule cells. The toolbox of criteria that we have investigated (in male and female mice) can be useful both for anatomical and functional experiments, and relies on the quantitative study of neuronal somatic/nuclear morphology, location and developmental profile, expression of specific molecular markers (GAD67, reelin, COUP-TFII, calretinin, and p73), single cell anatomy, and electrophysiological properties. We conclude that Cajal–Retzius cells are small, non-GABAergic neurons that are tightly associated with the hippocampal fissure (HF), and that, within this area of interest, selectively express the proteins p73 and calretinin. We highlight the dangers of using markers such as reelin or COUP-TFII to identify Cajal–Retzius cells or GABAergic interneurons because of their poor specificity. Lastly, we examine neurons of the postnatal hippocampal molecular layers and show cell type-specific differences in their dendritic/axonal morphologies and density distributions, as well as in their membrane properties and spontaneous synaptic inputs. These parameters can be used to distinguish biocytin-filled and/or electrophysiologically recorded neurons and should be considered to avoid interpretational mistakes.

## Significance Statement

The unambiguous identification of specific neuronal types in brain circuits is critical for a correct interpretation of experimental data. Cajal–Retzius cells of the molecular layers of the postnatal hippocampus are intermingled with other neurons, such as GABAergic interneurons and semilunar granule cells. Although the presence of Cajal–Retzius cells in the hippocampus is commonly acknowledged at early developmental stages, it is often overlooked at later points of maturation. This lack of attention may increase the risk of misclassification of Cajal–Retzius cells for GABAergic interneurons or other cell types and may lead to interpretative mistakes. Here, we provide a set of simple cell type-specific criteria for their unambiguous identification. Lastly, we also warn against equivocal criteria that may lead to erroneous classifications.

## Introduction

The ability to identify and distinguish cellular populations is essential for the correct interpretation of experimental data. In fact, since the description of the rich cellular diversity of the central nervous system by Ramon y Cajal (1899), it has become clear that the study of the morpho-functional properties of specific neurons and the rules governing their inter-connectivity (for review, see  Klausberger and Somogyi, 2008; Pelkey et al., 2017; Cembrowski and Spruston, 2019) are essential for a mechanistic understanding of the brain during physiologic or pathologic conditions (Markram, 2006; Maccaferri, 2011a,b; Paz and Huguenard, 2015; Kitamura et al., 2017; Soltesz and Losonczy, 2018).

Here, we address this general issue within the framework of the neuronal networks of the molecular layers of the hippocampus, which are involved in cognitive functions (Basu and Siegelbaum, 2015). These layers receive extra-hippocampal afferent inputs from various regions such as the thalamus and other subcortical areas, but most prominently from the entorhinal cortex (Amaral and Lavenex, 2007). These extra-hippocampal synaptic inputs are integrated by a complex local network and channeled onto the apical tufts of pyramidal cells in the cornu ammonis (CA) subfields (in stratum lacunosum-moleculare, see: Khazipov et al., 1995; Vida et al., 1998; Dvorak-Carbone and Schuman, 1999; Ang et al., 2005; Kitamura et al., 2014; Yamamoto and Tonegawa, 2017) and onto the dendrites of granule cells (in the dentate gyrus molecular layer, see: Sloviter, 1991; Scharfman, 1992; Buckmaster and Schwartzkroin, 1995; Sik et al., 1997; Calixto et al., 2008; Armstrong et al., 2011; Hsu et al., 2016; Mircheva et al., 2019). This local network includes microcircuits formed by glutamatergic Cajal–Retzius cells (Anstötz et al., 2016, 2018a,b,c) and by a heterogeneous population of GABAergic interneurons (Freund and Buzsáki, 1996). In addition, excitatory semilunar cells can be found in the molecular layer of the dentate gyrus (Williams et al., 2007).

Unfortunately, the distinction between these cell types, whose soma is located in the molecular layers, has not always been straightforward in the literature because of various reasons. First, some molecular markers used to identify Cajal–Retzius cells and/or GABAergic interneurons either lack specificity or have yielded contrasting experimental results. Second, Cajal–Retzius cells have been classically described as transient neurons that undergo apoptosis and virtually disappear in rodents shortly after birth. Although this observation is valid for Cajal–Retzius cells of the neocortex (Chowdhury et al., 2010), its generalization to the hippocampus may be problematic, as their density reduction is only partial (Anstötz et al., 2016, 2018a; Ledonne et al., 2016). Nevertheless, the persistence of Cajal–Retzius cells in the postnatal hippocampus is rarely taken into consideration by electrophysiological studies, such as recording from anatomically-unidentified cells of the molecular layers or when applying local stimulation with field electrodes.

Here, our two main goals are to highlight the importance of these general issues to the broader neuroscience community and provide, as a solution, a set of easily implementable criteria for morpho-functional studies that allow a clear distinction between Cajal–Retzius cells and other neuronal populations of the hippocampal molecular layers.

## Materials and Methods

### Ethical approval

All experimental procedures used in this study were approved by the Institutional Animal Care and Use Committee of Northwestern University and are in compliance with animal guidelines provided by the National Institutes of Health.

### Animals

Tg(CXCR4-EGFP)CD73Gsat/Mmucd male (*n* = 24) and female (*n* = 23) mice (MMRRC catalog #015859-UCD, RRID:MMRRC_015859-UCD, henceforth referred to as “CXCR4-EGFP mice” or simply “mice”) of different ages (between P7 and P60) were used for this study. All animals were housed with a 12/12 h light/dark cycle with food and water *ad libitum* before the experiments.

### Histologic analysis

CXCR4-EGFP mice were anesthetized by intraperitoneal injection of Euthasol (calculated to yield a dose of pentobarbital of 300 mg/kg of bodyweight), and perfused with 0.9% NaCl saline followed by 4% paraformaldehyde (PFA) in 0.1 M phosphate buffer (PB), pH 7.4. After perfusion, brains were extracted from the skull and transferred in fixative solution at 4°C for at least 24 h. Transversal hippocampal sections were cut serially at 50 μm thickness on a Leica VT 1000 vibratome and collected free-floating in 0.01 M PBS.

### Immunohistochemistry

Slices were preincubated free-floating in a blocking solution containing 5% normal goat serum (NGS), 1% bovine serum albumin (BSA) and 0.2% Triton X-100 in PBS for 1 h at room temperature (RT). Sections were then incubated free-floating in the same solution containing the primary antibodies (all 1:500) at 4°C overnight. The following primary antibodies were used for this study: rabbit anti-p73 (Abcam catalog #ab40658, RRID:AB_776999), rabbit anti-CoupTF2 (Millipore, ABE1426), goat anti-calretinin (Millipore catalog #AB1550, RRID:AB_90764), with mouse anti-GAD67 (Millipore catalog #MAB5406, RRID:AB_2278725) or mouse anti-reelin (Millipore catalog #MAB5364, RRID:AB_11212203) with rabbit anti-GAD67 polyclonal antibody (Abcam catalog #ab97739, RRID:AB_10681171). Furthermore, we used rabbit anti-Iba1 polyclonal antibody (Wako catalog #019-19741, RRID:AB_839504), rabbit anti-GFAP polyclonal antibody (Atlas Antibodies catalog #HPA056030, RRID:AB_2683015) with guinea pig anti-doublecortin (DCX) polyclonal antibody (Millipore catalog #AB2253, RRID:AB_1586992), and rabbit anti-p73 with mouse anti-NeuN (Abcam catalog #04224).

Slices were then washed 3 × 15 min with fresh PBS. Then slices were incubated free-floating in a solution of 5% NGS, 1% BSA in PBS, containing secondary antibodies (all 1:500) at RT for 1 h. The following secondary antibodies were used: Alexa Fluor 568 goat anti-rabbit IgG (Thermo Fisher Scientific catalog #A-11036, RRID:AB_10563566), Alexa Fluor 568 goat anti-mouse IgG (Thermo Fisher Scientific catalog #A-11004, RRID:AB_2534072), Alexa Fluor 568 donkey anti-mouse IgG (Thermo Fisher Scientific catalog #A10037, RRID:AB_2534013), Alexa Fluor 594 goat anti-guinea pig IgG (Thermo Fisher Scientific catalog #A-11076, RRID:AB_2534120) Alexa Fluor 647 goat anti-rabbit IgG (Thermo Fisher Scientific catalog #A32733, RRID:AB_2633282), Alexa Fluor 647 goat anti-mouse IgG (Thermo Fisher Scientific catalog #A28181, RRID:AB_2536165).

Slices were washed again 3 × 15 min with fresh PBS, while the second washing step contained DAPI (1:100,000, Life Technologies, #62249) to achieve fluorescent nuclear counterstaining. Slices were then mounted and coverslipped individually using Mowiol mounting medium.

### Confocal microscopy

Confocal microscopy image stacks were captured using a Leica SP8 confocal microscope and Nikon A1R. For images covering the entire hippocampal section, a 10× lens was used to acquire Z-stacks with a 5 µm interval at a resolution of 750 nm per pixel. For high-magnification images, a 63× lens was used to acquire Z-stacks with a 1 µm interval at a resolution of 120 nm per pixel. Multichannel fluorescence images were saved individually for analysis and merged together for co-localization studies using the Leica LAS AF or Nikon NIS Elements software suite.

### Analysis of immunoreactivity co-localization

To calculate the relative fraction of neurons with a specific set of molecular markers, co-localization of immunoreactivity was assessed at high-magnification stacks for different regions of the hippocampal molecular layers surrounding the hippocampal fissure (HF). Co-localization was identified using a line histogram drawn through a single neuron. A co-localization was noted if single peak histograms in both channels were either overlaying (in the same *z*-axis plane) or if a bimodal histogram in one channel surrounded a single peak histogram in the other channel (cytosolic marker surrounding a nuclear marker). The analysis was performed using the Leica LAS AF Lite software suite. In this study, “the vicinity of the HF” is defined as a region spanning a radial distance of ±125 µm to the HF because within this range 95% of the Cajal–Retzius cells (in a given frame) can be captured.

### Quantification of neuronal densities

Confocal microscopy stacks covering the entire hippocampal section were imported into a Neurolucida 11 software suite. Cajal–Retzius cells and GABAergic interneurons were identified and marked by their EGFP expression or GAD67 immunoreactivity, respectively. Then, relevant hippocampal borders were outlined. For these measurements, the entire molecular layers adjacent to the HF were chosen as the region of interest and cellular densities were calculated as line densities (as described in Anstötz et al., 2016).

### Generation of spatial density plots

2D maps of CR cell and IN densities were constructed using the raw data obtained by Neurolucida containing the exact spatial information of counted cells. The absolute spatial information of every CR cell was converted into a relative position using hippocampal fix-points (pole and split-point of the HF; medial and lateral curvature of the dentate gyrus; pial ending of the infrapyramidal blade of the dentate gyrus, see Anstötz et al., 2016). These normalized positions were plotted into an outline of a representative hippocampal formation. The number of neurons was measured in a 50 × 50 μm grid, yielding a raw density map. The data of this “region-specific density” plots were processed and visualized as a Contour-Plot in Origin 2019b. The lower bound of the scale was set to 0, the upper bound to the maximum density.

The acquired Neurolucida files containing the exact spatial information of every counted cell were analyzed using a custom script written in Visual Basic. For every counted neuron, the shortest distance between the center of its soma and the HF (a line perpendicularly connecting the neuron center to the HF) was calculated.

For the nearest neighbor analysis, the distances between the somatic centers of the neuron of interest and all other cells of the same type were calculated, and the shortest distance was used to identify the nearest neighbor.

To generate a simulated population of neurons that are uniformly distributed, neurons within the borders of a representative outlined hippocampus were plotted using a random generator. The number of generated neurons was set to match the densities of measured values. The line densities and nearest neighbor distances were calculated as explained above in this section (Extended Data Fig. 4-1).

### Electrophysiological methods

Acute hippocampal slices were prepared as follows. First, animals were deeply anesthetized with isoflurane and then decapitated. The brain was removed and transferred in a chilled modified artificial CSF (ASF) containing the following: 130 mM NaCl, 24 mM NaHCO_3_, 3.5 mM KCl, 1.25 mM NaH_2_PO_4_, 1 mM CaCl_2_, 2 mM MgSO_4_, and 10 mM glucose, saturated with 95% O_2_, 5% CO_2_ (pH 7.4). A vibratome (Leica VT 1200 S) was used to cut transverse sections (350 μm thickness), which were then incubated at 34–35°C for ∼ 30 min and then stored at RT in ACSF of the following composition: 130 mM NaCl, 24 mM NaHCO_3_, 3.5 mM KCl, 1.25 mM NaH_2_PO_4_, 2 mM CaCl_2_, 1 mM MgSO_4_, and 10 mM glucose, saturated with 95% O_2_, 5% CO_2_ (pH 7.4). When required, slices were transferred to a direct microscope (Scientifica SciScope) with oblique illumination optics and an infrared camera system (Zyla 4.2, Andor Technology). Cells were identified by their location and EGFP expression using a 60× IR water immersion objective and a LED (460-nm wavelength) light source (Prizmatix). Slices were superfused with preheated ACSF maintained at a constant temperature (29–31°C) by a temperature controller (TC-324B, Warner Instruments). Electrodes were pulled from borosilicate glass capillaries (Prism FLG15, Dagan Corporation) and had a resistance of 3–5 MΩ when filled with the appropriate internal solution, as reported below in the “Pipette solutions” section. Recordings were performed using a Multiclamp 700 amplifier (Molecular Devices). Analog signals were filtered at 3 kHz and digitized at 20 kHz (voltage clamp) or 50 kHz (current clamp) using a Digidata 1550B and the Clampex 10 program suite (Molecular Devices). Access resistance was compensated in current-clamp configuration with a bridge circuit. Membrane resistance was calculated in voltage clamp at a holding potential of –60 mV with a 1 s, –10 mV voltage step (only for experiments with pipette solution containing K-methylsulfonate).

### Pipette solutions

Current-clamp recordings from neurons were performed using the following intracellular solution: 125 mM K-methylsulfate, 10 mM NaCl, 0.3 mM GTP-Na, 4 mM ATP-Mg, 16 mM KHCO_3_, and mM 0.3–0.5% biocytin, equilibrated with 95% O_2_, 5% CO_2_ (pH 7.3) and 0.2–0.4% biocytin.

Voltage-clamp recordings from neurons used pipettes filled with the following solution: 125 mM Cs-methanesulfonate, 0.3 mM GTP-Na, 4 mM ATP-Mg, 16 mM KHCO_3_, 10 mM QX314-Cl, and 0.2–0.4% biocytin, equilibrated with 95% O_2_, 5% CO_2_ (pH 7.3) and 0.2–0.4% biocytin.

### Recovery of biocytin-filled cells and reconstructions

Biocytin-filled neurons were fixed in 4% PFA in 0.1 M PB at 4°C for at least 24 h. Endogenous peroxidase activity was quenched with a 3% H_2_O_2_ solution for 15 min. Sections were incubated overnight at 4°C in avidin-biotinylated-HRP complex (Vectastain ABC Elite kit) with 0.1% Triton X-100 in PB, followed by a peroxidase reaction with DAB tetrahydrochloride as a chromogen. Cells were revealed by adding 0.025% H_2_O_2_, and the reaction was stopped when dendritic and axonal processes were clearly visible under light microscopy examination. After several washing steps in 0.1 M PB, slices were postfixed with 0.1% OsO_4_ in PB (1–2 min), and then mounted on slides with Mowiol (Hoechst AG). Cells were reconstructed using a using a NEUROLUCIDA-based station and software. *Post hoc* biometric data were generated by NERUOLUCIDA Explorer software. Sholl analysis was performed with a 50 μm starting radius and a 50 μm interval. Dendritic and axonal density-plots were generated as described in Anstötz et al. (2016). Briefly, neurons were aligned at their soma center and with respect to their orientation in the hippocampus. The dendrites and axons were then plotted in a 50 × 50 µm Cartesian grid and total length of each segment within each grid box was calculated. The resulting raw density map was illustrated using a contour-plot with Origin 2019b.

### Generation of axonal and dendritic density plots

Reconstructed neurons were merged and aligned the center of their soma. The combined reconstructed neurons were placed in a Cartesian grid and the average length of each neuronal segment within grid-boxes of 25 × 25 µm was calculated, yielding a raw density matrix. To obtain the axo-dendritic overlap, the axonal matrix was multiplied by the dendritic matrix and then normalized to the sum of all values. The matrices were then transferred to OriginPro 2019b (Origin Lab) to create a contour plot.

### Statistical methods

Statistics were performed using a Mann–Whitney *U* test comparing two groups or ranked ANOVA comparing multiple groups/categories with LSD *post hoc* tests in Origin 2019b. Level of significance for individual tests was chosen as *p* < 0.05. The level of significance in the figures is indicated as follows: n.s., *p* > 0.05, *p < 0.05, **p < 0.01, ***p < 0.001. Values in the text are given as mean ± standard error. Box plots in the illustrations indicate the median (middle dash), mean (circle) the lower and upper quartile (box borders), and minimum and maximum values (whiskers).

## Results

**Figure 1.**
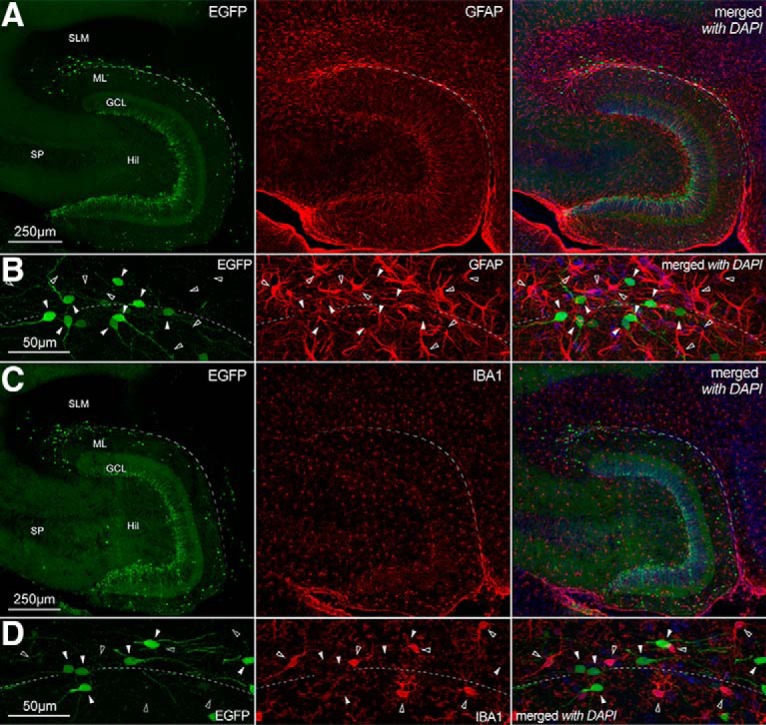
EGFP-expressing cells located in the molecular layers of the CXCR4-EGFP mouse are not immunoreactive for astrocytic or microglial markers. ***A***, Low-magnification images of the hippocampal dentate gyrus of the CXCR4-EGFP mouse. Left, EGFP expression (green). Middle, GFAP immunoreactivity (red). Right, Previous images superimposed with additional DAPI counterstaining (blue). SLM, stratum lacunosum-moleculare; ML, molecular layer; GCL, granule cell layer; Hil, hilus; SP, stratum pyramidale. ***B***, Higher-magnification view of the region in proximity to the HF (dotted line); left, middle, and right as in ***A***. Notice the total lack of co-localization between the EGFP and GFAP signals. Filled arrowheads indicate EGFP-expressing somata, whereas empty arrowheads mark the position of GFPA-labeled cells. ***C***, ***D***, Identical organization as in ***A***, ***B***, respectively, but immunoreactivity is shown for the microglial marker IBA1. Notice that EGFP-positive cells are not IBA1-immunoreactive.

**Figure 2.**
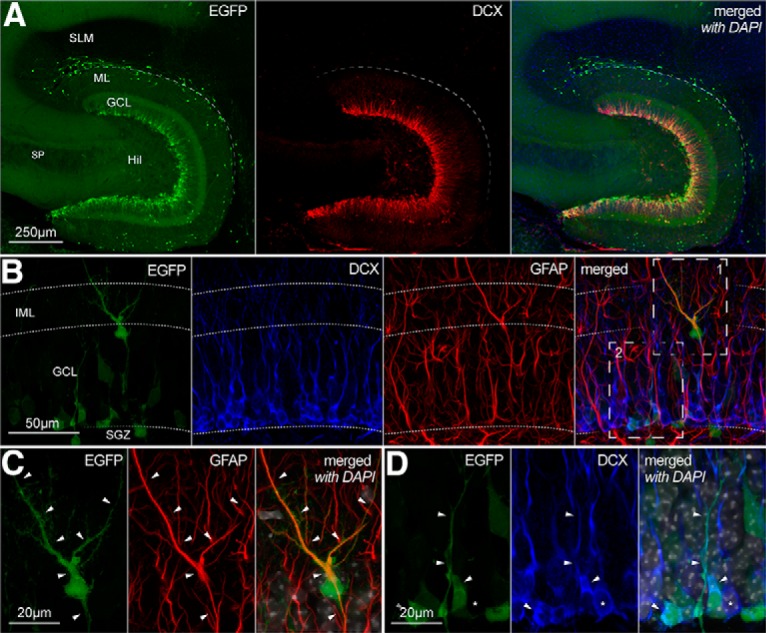
EGFP expression in the neurogenic niche of the CXCR4-EGFP mouse. ***A***, Low-magnification images of the hippocampal dentate gyrus showing EGFP expression (left, green), DCX immunoreactivity (middle, red), and the two signals superimposed with DAPI (blue) counterstain (right). SLM, stratum lacunosum-moleculare; ML, molecular layer; GCL, granule cell layer; Hil, hilus; SP, stratum pyramidale. ***B***, Higher magnification reveals EGFP expression (green, left) in DCX-negative (blue, middle left), GFAP-positive (red, middle right) cells with the soma in the GCL and processes extending to the inner molecular layer (IML) as well as in DCX-positive, GFAP-negative cells, in the subgranular zone (SGZ). The right panel shows all the previous signals superimposed. Dotted lines mark the border between different layers. The boxes in the right panel are shown in more detail in ***C***, ***D***, for EGFP expression (left, green), GFAP/DCX immunoreactivity (middle, red and blue, respectively), and with the signal superimposed and additional DAPI counterstaining (white, right). Filled arrowheads mark the position of the soma and processes of the cells of interest for reference. Asterisk marks EGFP-negative, DCX-positive cell.

Throughout this study, we have used a BAC transgenic animal (the CXCR4-EGFP mouse, see Methods for details) as a tool to reveal Cajal–Retzius cells of the molecular layers. We took advantage of this line, as we had already validated its specificity for Cajal–Retzius cells of hippocampal molecular layers and neocortical layer 1 in previous publications (Marchionni et al., 2010, 2012; Cosgrove and Maccaferri, 2012; Quattrocolo and Maccaferri, 2013; Anstötz et al., 2014, 2016, 2018b, 2019). In fact, although CXCR4 may be present on the membrane of both astrocytes and microglia (Tanabe et al., 1997), this does not drive the expression of EGFP in these non-neuronal cell types (within the molecular layers of the hippocampus). We confirmed this in Figure 1, where EGFP-expressing cells were found immunonegative, both for the astrocytic and microglial markers GFAP and IBA1, respectively. Furthermore, EGFP-labeled cells displayed the stereotypical and distinctive tadpole-like shape of hippocampal Cajal–Retzius neurons (similar to what described by Anstötz et al., 2016). In contrast, GFAP-expressing astrocytes showed a typical stellate appearance and IBA1-positive microglial cells were endowed with short and complex processes. From a total of 1673 GFAP-expressing cells 0% were EGFP-positive and no EGFP-expressing cell showed immunoreactivity for GFAP (*n* = 545 cells, *n* = 3 mice). No co-localization was observed between EGFP and IBA1 either (*n* = 950 IBA1-immunoreactive and *n* = 607 EGFP-positive cells examined, *n* = 3 mice). Similar results were observed in animals of different ages (P7, P30, P60; data not shown). Outside the molecular layers, we detected EGFP-expression in cells of the neurogenic niche thus confirming the results of Bhattacharyya et al. (2008; Fig. 2).

**Figure 3.**
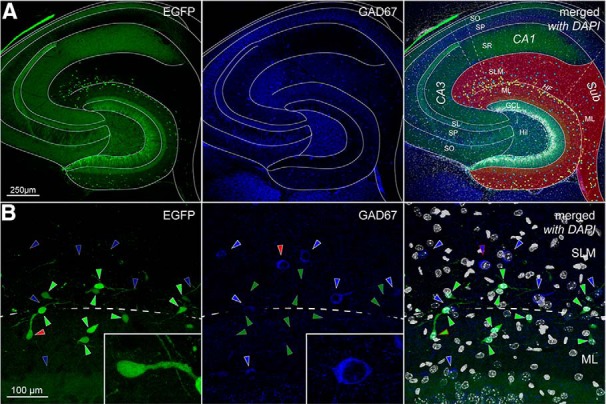
Overview of the distribution of EGFP-positive and GAD67-immunoreactive neurons in the CXCR4-EGFP mouse (P14). ***A***, left, Low-magnification fluorescence image of the hippocampus. Notice the numerous EGFP-labeled neurons localized in the molecular layers on either side of the HF. Middle, Immunoreactivity for GAD67 in the same section. Notice the more scattered distribution of labeled cells within all hippocampal layers. Right, Overlap of the previous images with nuclear DAPI staining. Specific layers and areas are indicated as follows: SO, stratum oriens; SP, stratum pyramidale; SL, stratum lucidum; SLM, stratum lacunosum-moleculare; HF, hippocampal fissure; ML, molecular layer; GCL, granule cell layer; Hil, hilus; CA3, cornu ammonis 3; CA1, cornu ammonis 1; Sub, subiculum. Dashed lines indicate the borders between the CA3 and CA1 subfields and of the subiculum. Hippocampal molecular layers (MLs) are highlighted in red. Circles indicate the position of EGFP-positive cells (green/white) and GAD67-positive cells (blue/white). ***B***, Same experimental setup as in ***A***, but observed at higher magnification. The dotted line indicates the HF. Notice the lack of overlap between the EGFP and GAD67 signals. Arrowheads indicate EGFP-positive Cajal–Retzius cells (green) and GAD67-positive interneuron (blue). Insets show the neurons indicated by red arrowheads at higher magnification.

Next, we addressed the claim, which has been proposed in several papers (Imamoto et al., 1994; Pesold et al., 1998; Yu et al., 2014) and review articles (Sarnat and Flores-Sarnat, 2014), that Cajal–Retzius cells (or a subpopulation of the main class, see distinction between “Cajal–Retzius cells proper” and “Cajal–Retzius cells” discussed in the review by DeFelipe et al., 2013) are GABAergic interneurons. Of course, a conclusive determination of this point is critical for the correct interpretations of experimental results. When we compared the EGFP expression of molecular layer neurons (which identifies them as Cajal–Retzius cells in the CXCR4-EGFP mouse) versus GAD67 immunoreactivity (which is considered a pan-GABAergic marker for interneurons) we observed two distinct, non-overlapping, populations of cells (Fig. 3, P14 animal). From a total of 631 neurons, EGFP expression was found in 350 cells compared to 280 neurons immunoreactive for GAD67. Only a single neuron in the entire sample was apparently labeled for both markers, which suggests the occurrence of an artifactual overlap of fluorescence. Similar results were obtained in animals of different ages (P7, P30, P60; Table 1). Thus, our data are in line with the interpretation that Cajal–Retzius cells do not belong to the general class of GABAergic neurons and fit well with previous work in the literature (in contrast to what quoted at the beginning of this paragraph) reporting their lack of expression of GABAergic markers (del Río et al., 1995; Soda et al., 2003; Hevner et al., 2003; Anstötz et al., 2016). Furthermore, they corroborate previous functional studies showing that the monosynaptic responses observed on target cells following the stimulation of Cajal–Retzius cells are mediated by AMPA-type glutamate receptors (Quattrocolo and Maccaferri, 2014; Anstötz et al., 2016).

**Table 1.** Developmental profile of the relative proportions of EGFP-expressing and GAD67-expressing neurons of the hippocampal molecular layers in the CXCR4-EGFP mouse

Measurement	Age			
	P7	P14	P30	P60
EGFP-positive	1541	1381	1079	870
GAD67-positive	652	1114	870	1041
EGFP/GAD67-positive	1	1	0	0

Notice that EGFP- and GAD67-labeled neurons remain two distinct populations throughout P7–P60. Results obtained from three mice per age.

**Figure 4.**
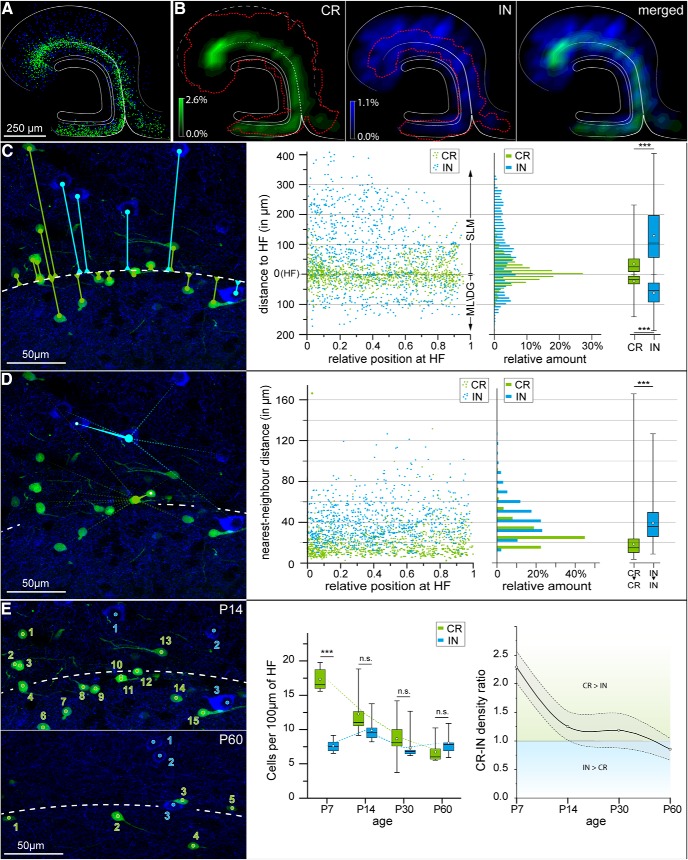
Spatiotemporal distribution of EGFP-labeled Cajal–Retzius cells versus GAD67-immunoreactive interneurons of the molecular layers. ***A***, Summary plot showing the normalized location of Cajal–Retzius cells and GABAergic interneurons of the hippocampal molecular layers. Data from *n* = 3 animals (P14), *n* = 690 Cajal–Retzius cells and *n* = 713 interneurons. Schematic lines indicate the granule cell layer, HF, and borders of the infra-pyramidal blade of the dentate gyrus and subicular complex. ***B***, left, Fractional density plot of Cajal–Retzius cells (green) and 5% maximum density area for interneurons (dotted red contour). Middle, Similar to the left panel, but with fractional density of interneurons (blue) and the 5% maximum density area for Cajal–Retzius cells limited by the dotted red line. Notice the more restricted area occupied by EGFP-labeled Cajal–Retzius cells compared to GAD67-immunoreactive interneurons. Right, Left and middle panels superimposed. ***C***, Distances of Cajal–Retzius and GABAergic interneurons from the HF. Left, Microscopic field with measurements of the shortest distances between the soma of Cajal–Retzius cells and interneurons (green and blue lines and circles, respectively) from the HF (white dotted line). Middle, Summary scatterplot for measurements from Cajal–Retzius cells (CR, green dots) and GABAergic interneurons (IN, blue dots). HF, hippocampal fissure. The abscissa indicates the relative position of the studied cell from the hippocampal pole (0) to the end of the HF (1). ML/DG, molecular layer of the dentate gyrus; SLM, stratum lacunosum-moleculare. Right (left panel), Relative distributions of the distances to the HF for the two neuronal populations (green, Cajal–Retzius cells; blue, interneurons). Notice the clear grouping of Cajal–Retzius cells in the vicinity of the HF compared to a more homogeneous distribution in the case of interneurons. Right (right panel), Summary box charts indicating the values of the distances from the fissure calculated separately for Cajal–Retzius cells and interneurons of stratum lacunosum-moleculare (top) and of the molecular layer of the dentate gyrus (bottom). Notice, in both cases, the shorter distances of Cajal–Retzius cells. ***D***, Cajal–Retzius cell are positioned in a non-random fashion. Left, Measurement of nearest neighbor distances between Cajal–Retzius cells and interneurons. The image shows examples for a Cajal–Retzius cell (green dot) and an interneuron (blue dot). The nearest neighbor (Cajal–Retzius cell to Cajal–Retzius cell and interneuron to interneuron) is identified in both cases by a white dot. The distance to the nearest neighbor is marked by the thick tapered lines, whereas distances to other cells are shown by faint dotted lines (green for Cajal–Retzius cells and blue for interneurons). The white dashed line indicates the HF. Middle, Summary graph for Cajal–Retzius cells (green dots) and interneurons (blue dots). The abscissa indicates the relative position from the hippocampal pole (0) to the end of the CA1 region (1). Right (left panel), Distribution histograms of the data shown in the scatterplot. Right (right panel), Summary box charts for nearest neighbor distances between Cajal–Retzius cells (green box), interneurons (blue box). Notice the much shorter distances for Cajal–Retzius cells. ***E***, left, Comparison of the linear densities of Cajal–Retzius cells and interneurons at different postnatal stages (P14, top panel; P60, bottom panel). Cajal–Retzius cells and interneurons are counted in green and blue, respectively. Middle, Summary boxplot for linear densities measured at P7, P14, P30, and P60. Notice the larger density of Cajal–Retzius cells progressively approaching the values measured for interneurons with brain maturation. Boxes have been slightly shifted to the left and right of the actual time points (P7, P14, P30, and P60) to avoid their superimposition. The connecting dotted lines are aligned to the actual time points. Right, Summary graph of the ratio of the densities of Cajal–Retzius cells over interneurons. Notice that at early developmental stages the density of Cajal–Retzius cells is more than double the one of the entire population of GAD67-positive cells in the same molecular layers. n.s., *p* > 0.05, ***p < 0.001.

If Cajal–Retzius cells and GABAergic interneurons were two separate neuronal populations, then we predicted that we might observe cell type-specificity in their spatial distribution. When this aspect was examined quantitatively, two main differences emerged. First, the localization of Cajal–Retzius cells was more tightly associated with the HF compared to interneurons (Fig. 4, P14 animal). Within stratum lacunosum-moleculare, the distance between the HF and EGFP neurons (34.43 ± 1.48 µm, *n* = 515) was shorter compared to GAD67-labeled cells (130.04 ± 3.67 µm, *n* = 676, *p* < 0.001). The same architectural principle was maintained in the molecular layers of the dentate gyrus (22.44 ± 0.88 µm for Cajal–Retzius cells, *n* = 641 vs 62.86 ± 2.41 µm for interneurons, *n* = 406, *p* < 0.001). Second, we observed that Cajal–Retzius cells had an apparent clustered-like/condensed distribution when compared to interneurons. The measured nearest neighbor distance between pairs of EGFP-positive cells was 18.70 ± 0.39 µm (*n* = 1156) versus 39.21 ± 0.62 µm in pairs of GAD67-expressing neurons (*n* = 932, *p* > 0.001). Similar measurements were obtained in animals of different ages (P7, P30, P60; Table 2). The possibility that this result depended on cell type-specific differences in densities was ruled out by comparing the average nearest neighbor distances against the average density for both neuronal populations with a model of a random equal distribution (Extended Data Fig. 4-1). Therefore, we think that the observed difference is most parsimoniously explained by a strong, postnatally maintained, chemoattractant influence of the chemokine CXCL12 (produced by cells investing the HF; Lu et al., 2002; Anstötz et al., 2019) on CXCR4-expressing Cajal–Retzius cells (Stumm et al., 2002; Marchionni et al., 2010). In fact, although CXCR4 is also critical for regulating the migration of GABAergic interneurons during prenatal stages (Stumm et al., 2003; Li et al., 2008; López-Bendito et al., 2008), its postnatal expression in the molecular layers appears to be restricted to Cajal–Retzius cells, which may explain the specificity of the labeling in the CXCR4-EGFP mouse for these neurons (Stumm et al., 2003; Tran et al., 2007; Kolodziej et al., 2008, Anstötz et al., 2019).

**Table 2.** Summary table of the distances and densities of Cajal–Retzius cells and interneurons measured in mice of different developmental stages (P7–P60, *n* = 3 animals for every age)

		Age				
Measurement	Unit	P7	P14	P30	P60	Cell type
Distance to HF ML - dentate gyrus	µm	20.29 ± 1.02	23.45 ± 0.88	21.46 ± 1.06	26.00 ± 1.03	CR
		74.82 ± 4.71	72.54 ± 2.99	72.52 ± 2.77	87.06 ± 3.3	IN
		27%	32%	30%	30%	CR/IN ratio
		219/120	641/300	522/223	435/278	#CR/#IN
						
Distance to HF SLM - cornu ammonis	µm	33.79 ± 1.89	34.49 ± 1.48	23.17 ± 1.26	26.43 ± 1.14	CR
		113.06 ± 5.49	129.71 ± 3.02	130.04 ± 3.67	112.26 ± 3.33	IN
		30%	27%	18%	24%	CR/IN ratio
		245/223	514/633	393/492	337/659	#CR/#IN
						
Nearest neighbor distance	µm	15.33 ± 0.46	18.71 ± 0.39	19.81 ± 0.43	22.80 ± 0.62	CR
		38.81 ± 1.24	39.61 ± 0.62	39.61 ± 0.68	39.99 ± 0.79	IN
		40%	47%	50%	57%	CR/IN ratio
		464/343	1155/933	945/715	772/937	#CR/#IN
						
Density at HF	Cells/100 µm	17.33 ± 0.63	12.27 ± 1.02	8.64 ± 0.79	6.74 ± 0.53	CR
		7.62 ± 0.33	9.89 ± 0.5 + 6	7.34 ± 0.68	8.05 ± 0.55	IN
		227%	124%	118%	84%	CR/IN ratio
		770/326	1381/1114	1079/870	870/1041	#CR/#IN

Compare to results illustrated in Figure 2. CR: EGFP-identified Cajal–Retzius cells, in: GAD67-immunoreactive interneurons. #CR and #IN indicate the number of the different cell types used for the analysis.

10.1523/ENEURO.0516-19.2019.f4-1 Extended Data Figure 4-1 Nearest neighbor distances of Cajal–Retzius cells and GABAergic interneurons compared to a simulated a random equal distribution (R.E.D.). ***A***, Plot of Cajal–Retzius cells (*n* = 461), interneurons (*n* = 511), and R.E.D. cells (*n* = 500), in a normalized model of the hippocampus. ***B***, Plot of the linear cell density of Cajal–Retzius cells (*n* = 45 slices) and interneurons (*n* = 33 slices) against their average nearest neighbor distance (per slice). The R.E.D. perfectly follows an allometric fitted function (red line, *y* = 111.83×^−0.54^; *R* ^2^ = 1), based on a simulation with *n* = 5× values (25 iterations each). Notice that the GABAergic interneurons are closely associated with the function of the R.E.D. suggesting their equal distribution in the hippocampal molecular layers. The distribution of Cajal–Retzius cells is shifted downwards, suggesting a clustered distribution. Selection of data points shown in ***A*** have a black outline. Download Figure 4-1, TIF file.

Lastly, we noticed an additional striking difference in the developmental profiles of the densities of EGFP-expressing Cajal–Retzius cells (at P7: 17.33 ± 0.58, P14:12.27 ± 0.96, P30: 8.64 ± 0.91, P60: 6.74 ± 0.50; in cells per 100 µm of HF; see also Table 2) versus GAD67-labeled interneurons (at P7: 7.61 ± 0.31, P14:9.89 ± 053, P30: 7.34 ± 0.64, P60: 8.052 ± 0.52; in cells per 100 µm of HF, *p* < 0.001). In fact, in contrast to the early postnatal sharp density decrease of EGFP-expressing cells, the density of GABAergic interneurons remained fairly constant.

**Figure 5.**
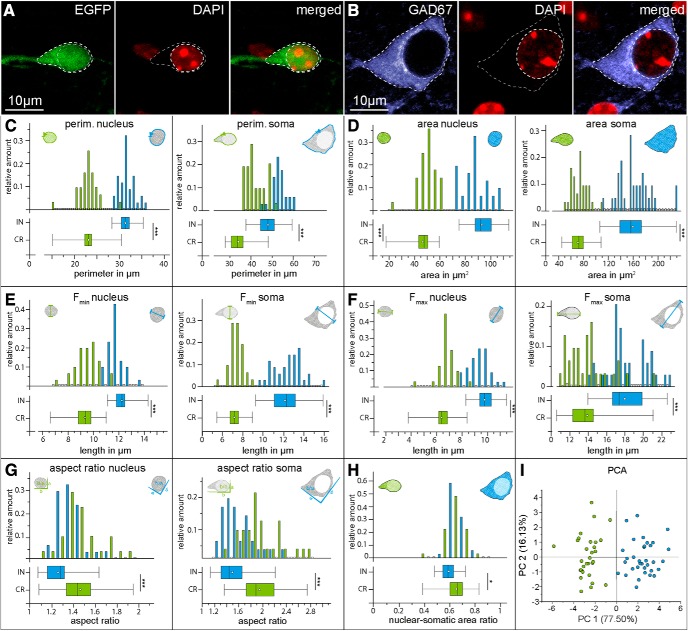
Comparison of biometric morphologic parameters between Cajal–Retzius cells and interneurons. ***A***, Regions of interest (soma and nucleus: white dotted lines) and markers in individual Cajal–Retzius cells (three leftmost panels: EGFP, DAPI, and superimposed) and ***B***, interneurons (three rightmost panels: GAD67, DAPI, and superimposed). ***C****–****H*,** Comparisons of the distributions for the measured parameters (top graphs) in Cajal–Retzius cells and interneurons (green and blue, respectively) and their summary box charts (bottom insets). Notice the overall smaller size of Cajal–Retzius cells compared to interneurons; (***C***) nuclear (left) and somatic (right) perimeter, (***D***) nuclear (left) and somatic (right) area, (***E***) nuclear (left) and somatic (right) minimal Feret diameter (F_min_), (***F***) nuclear (left) and somatic (right) maximal Feret diameter (F_max_), (***G***) nuclear (left) and somatic (right) aspect ratios, (***H***) nuclear/somatic areas ratio, (***I***) principal component analysis of all the measured parameters. Notice the clear separation in clusters between Cajal–Retzius cells (green circles) and interneurons (blue circles). *p < 0.05, ***p < 0.001.

Next, we examined differences in morphologic biometrical indexes between Cajal–Retzius cells and GABAergic interneurons located in the molecular layers. We measured eleven parameters and built their frequency distributions (Fig. 5). In all cases, the comparison of parameters measured (P14 animal) in EGFP-expressing Cajal–Retzius (*n* = 31) versus GAD67-immunoreactive (*n* = 34) interneurons revealed significant differences (see also Table 3 for data obtained at P7, P30, and P60). In particular, the distribution of the nuclear (Cajal–Retzius cells: 46.32 ± 1.71 vs 93.04 ± 1.84 µm^2^ in interneurons, *p* < 0.001) and somatic areas (Cajal–Retzius cells: 71.58 ± 2.38 vs 159.37 ± 4.57 µm^2^ in interneurons *p* < 0.001) appeared well separated and with minimal overlap between the two cell populations. When principal component analysis was performed on the eleven parameters, plotting the data according to the first two principal components was sufficient to generate two well separated clusters of cells.

**Table 3.** Summary table of the values for the various morphometric parameters measured as in Figure 3 for mice of different ages (P7–P60, *n* = 3 animals for every age)

		Age								
Measurement	Unit	P7		P14		P30		P60		Cell type
		Nucleus	Soma	Nucleus	Soma	Nucleus	Soma	Nucleus	Soma	
Perimeter	µm	28.77 ± 1.59	40.25 ± 1.62	25.45 ± 0.49	34.47 ± 0.83	24.12 ± 0.56	32.54 ± 0.71	23.92 ± 0.41	31.72 ± 0.79	CR
		36.06 ± 1.16	49.74 ± 1.55	34.83 ± 0.33	48.53 ± 0.83	33.64 ± 0.91	47.74 ± 0.54	31.12 ± 0.65	42.56 ± 1.03	IN
		79.8%	80.9%	73.1%	71.0%	71.7%	68.2%	76.9%	76.9%	CR/IN ratio
										
Area	µm²	53.08 ± 3.05	85.08 ± 5.84	46.32 ± 1.43	71.58 ± 2.38	42.12 ± 2.18	68.36 ± 3.01	38.31 ± 1.08	55.26 ± 1.93	CR
		94.74 ± 4.38	157.8 ± 8.71	93.04 ± 1.87	159.37 ± 4.64	94.03 ± 1.99	121.32 ± 2.84	70.63 ± 2.59	116.42 ± 4.11	IN
		56.0%	53.9%	49.8%	44.9%	44.8%	56.4%	54.2%	47.5%	CR/IN ratio
** **	** **	** **	** **	** **	** **	** **	** **	** **	** **	
Feret max	µm	10.31 ± 0.22	16.91 ± 0.67	9.31 ± 0.18	13.93 ± 0.42	10.01 ± 0.32	14.27 ± 0.55	9.41 ± 0.27	13.21 ± 0.46	CR
		13.08 ± 0.42	18.5 ± 0.64	12.26 ± 0.12	18.05 ± 0.38	12.08 ± 0.37	16.21 ± 0.41	11.54 ± 0.35	16.54 ± 0.57	IN
		78.8%	91.4%	75.9%	77.2%	82.9%	88.0%	81.5%	79.9%	CR/IN ratio
** **	** **	** **	** **	** **	** **	** **	** **	** **	** **	
Feret min	µm	6.55 ± 0.31	7.32 ± 0.34	6.42 ± 0.14	7.21 ± 0.13	6.21 ± 0.12	7.24 ± 0.21	5.31 ± 0.17	6.07 ± 0.19	CR
		9.34 ± 0.26	12.03 ± 0.45	9.78 ± 0.15	12.19 ± 0.25	9.11 ± 0.18	11.41 ± 0.22	10.03 ± 0.25	7.99 ± 0.19	IN
		78.8%	91.4%	75.9%	77.2%	82.9%	88.0%	81.5%	79.9%	CR/IN ratio
** **	** **	** **	** **	** **	** **	** **	** **	** **	** **	
Aspect ratio	-	1.65 ± 0.09	2.36 ± 0.08	1.47 ± 0.04	1.95 ± 0.06	1.81 ± 0.09	2.08 ± 0.04	1.83 ± 0.1	2.25 ± 0.12	CR
		1.41 ± 0.04	1.57 ± 0.07	1.26 ± 0.02	1.5 ± 0.04	1.32 ± 0.02	1.55 ± 0.07	1.46 ± 0.06	1.67 ± 0.07	IN
		117.0%	150.3%	116.7%	130.0%	137.1%	134.2%	125.3%	134.7%	CR/IN ratio
										
*n*		23/20		31/34		32/31		25/25		#CR/#IN

CR: EGFP-identified Cajal–Retzius cells, in: GAD67-immunoreactive interneurons. #CR and #IN indicate the number of the different cell types used for the analysis.

These results fit well with the simple interpretation that Cajal–Retzius cells are, overall, smaller than GABAergic interneurons and can be readily distinguished by their size. Therefore, this knowledge could be easily used as a first approach for preselecting cells located in the hippocampal molecular layers (during electrophysiological recordings on living slices) or when studying new molecular markers (for immunohistochemical experiments). We then attempted to resolve interpretative ambiguities regarding the specificity of molecular markers commonly used for the identification of Cajal–Retzius cells and GABAergic interneurons. In particular, we focused on the glycoprotein reelin (Armstrong et al., 2019), the nuclear transcription factor COUP-TFII (Wang et al., 1989), the calcium-binding protein calretinin (Yamazaki et al., 2004) and the tumor protein p73 (Tissir et al., 2009). We decided to quantify and compare the relative fractions of Cajal–Retzius cells and GABAergic interneurons (in the vicinity of the HF, see Materials and Methods for the selection of the regions of interest) that were immunolabeled by these markers (*n* = 3 animals, *n* = 4 slices per animal, P14).

**Figure 6.**
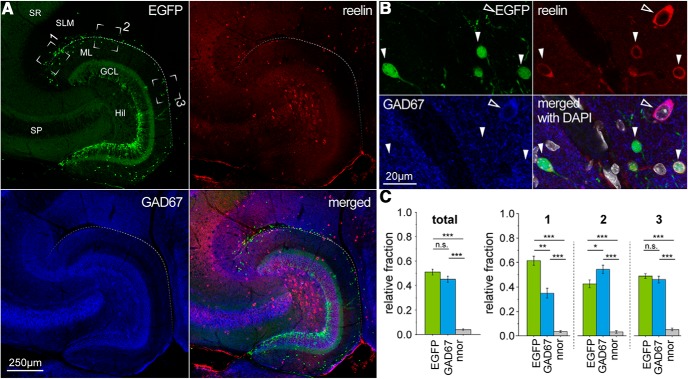
Reelin expression in the hippocampal molecular layers is not specific for Cajal–Retzius cells. ***A***, Low-magnification images of EGFP fluorescence (top left, green), reelin immunoreactivity (top right, red) and GAD67 immunoreactivity (bottom left, blue), and of all the signals superimposed (bottom right). Notice the presence of reelin-immunoreactive cells in all hippocampal layers. ***B***, Higher-magnification view reveals the presence of reelin both in EGFP-positive Cajal–Retzius cells and GAD67-expressing interneurons. EGFP expression (top left), reelin immunoreactivity (top right), GAD67 immunoreactivity (bottom left), and merged signals with DAPI (white) nuclear counterstaining (bottom right). Solid arrowheads indicate Cajal–Retzius cells; outlined arrowheads indicate GAD67-positive interneurons. ***C***, left, Summary plot of the relative fraction of reelin-expressing neurons that are positive for EGFP, GAD67, or neither EGFP nor GAD67 (nnor). Right, Regional subdivision of the data in the left panel for different regions of interest. 1, CA3 subfield; 2, CA1 subfield; 3, subiculum. n.s., *p* > 0.05, *p < 0.05, **p < 0.01, ***p < 0.001.

As shown in Figure 6, reelin was found to be expressed both by Cajal–Retzius cells and interneurons. From a total of *n* = 1184 reelin-expressing neurons examined at high magnification, *n* = 627 were classified as EGFP-positive/GAD67-negative (Cajal–Retzius cells), whereas *n* = 494 were found EGFP-negative/GAD67-positive (GABAergic interneurons) and we found that *n* = 43 did not express either EGFP or GAD67 (neither EGFP-positive Cajal–Retzius cells nor GAD67-positive GABAergic interneurons). Consistent with an especially high density of Cajal–Retzius cells at the “hippocampal pole” region (Anstötz et al., 2016; identified in Figure 6 as region of interest 1: RO1), reelin-immunoreactive cells in this area were predominantly EGFP-positive/GAD67-negative (61% vs 34% for EGFP-negative/GAD67-positive, *p* < 0.001). In the other areas that we measured (RO2 and RO3 along the HF corresponding to the CA1 subfield and the subiculum, respectively), the proportions of Cajal–Retzius cells and interneurons were more similar (for RO2: 42% vs 54%, *p* = 0.39 and for RO3: 49% vs 46%, *p* = 0.58). Overall, EGFP-expressing cells were consistently immunoreactive for reelin (100%, *n* = 647 EGFP-positive cells). Thus, our data confirm previous results that both Cajal–Retzius cells (Ogawa et al., 1995) and specific classes of interneurons of the molecular layers (neurogliaform cells, see Fuentealba et al., 2010) express reelin. More importantly, this outcome highlights the fact that reelin as a single marker is not sufficiently specific to discriminate between postnatal Cajal–Retzius cells and GABAergic interneurons. Similar results were obtained in animals of different ages (P7, P30, P60; data not shown), thus reinforcing our conclusion that the use of reelin as an exclusive marker for Cajal–Retzius cells in the postnatal brain should be abandoned. We also noticed the importance of examining cells at high magnification. In fact, the use of a lower gain to avoid saturation of the signal in strongly reelin-immunoreactive neurons outside the molecular layers (for example in the hilus, see Fig. 6) may impair the detection of more weakly labeled cells in the molecular layers. 

**Figure 7.**
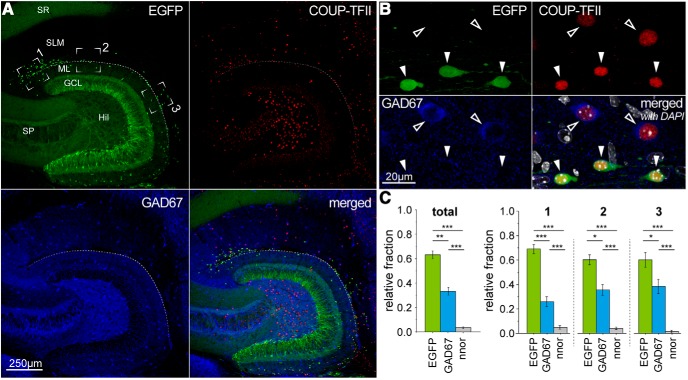
The nuclear transcription factor COUP-TFII is expressed both in Cajal–Retzius cells and interneurons of the molecular layers. ***A***, Low-magnification overview of the hippocampus tested for EGFP expression (top left, green), COUP-TFII immunoreactivity (top right, red), GAD67 immunoreactivity (bottom right, blue), and of all the signals superimposed (bottom right). ***B***, Cells of the molecular layers identified by their EGFP expression as Cajal–Retzius cells (top left, green) or GAD67-expressing interneurons (bottom left, blue) express COUP-TFII (top right, red). Bottom right, All signals superimposed with DAPI (white) nuclear counterstaining. Solid arrowheads indicate Cajal–Retzius cells; outlined arrowheads indicate GAD67-positive interneurons. ***C***, Left, Overall graph of the relative fraction of COUP-TFII-immunopositive cells that express EFGP, GAD67, or neither EGFP nor GAD67 (nnor). Right, Regional subdivision of the data in the left panel for different regions of interest. 1, CA3 subfield; 2, CA1 subfield; 3, subiculum. Notice that in any case, the largest fraction of COUP-TFII labeled neurons identifies Cajal–Retzius cells.

The second marker that we examined was the nuclear transcription factor COUP-TFII, which had been suggested to be specifically expressed, within the hippocampal molecular layers, by “putative neurogliaform cells” (Fuentealba et al., 2010). As shown by Figure 7, not only was COUP-TFII expressed, as expected, by GABAergic interneurons, but it was found also in Cajal–Retzius cells. Therefore, we concluded that COUP-TFII should not be considered a specific marker for neurogliaform cells. We also noticed, consistently with our results of Figure 4, the different somatic and nuclear sizes of the COUP-TFII-immunoreactive neurons, which suggests the presence of two distinct populations of cells. From a total of *n* = 571 COUP-TFII-positive cells, *n* = 364, were EGFP-positive/GAD67-negative, *n* = 185 were EGFP-negative/GAD67-positive and *n* = 36 were EGFP-negative/GAD67-negative (*p* < 0.001). Also, EGFP-expressing cells were consistently immunoreactive for COUP-TFII (98.6%, *n* = 618 EGFP-positive cells). These measurements indicate that Cajal–Retzius cells (and not GABAergic interneurons) are the predominant COUP-TFII-expressing population in the vicinity of the HF at all the developmental stages examined (P14: Fig. 7, and P7, P30, P60; data not shown).

**Figure 8.**
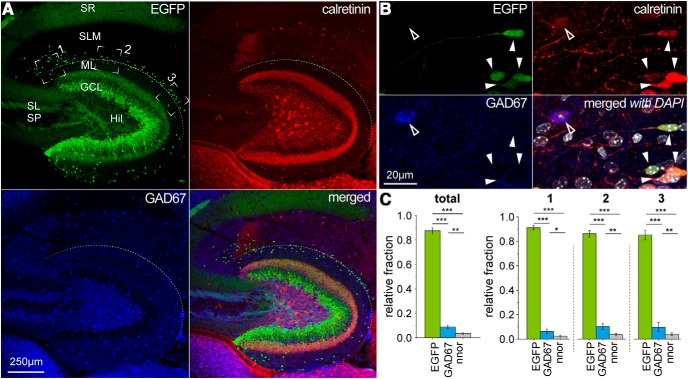
Immunoreactivity for the calcium binding protein calretinin in the molecular layers of the hippocampus is predominantly due to its expression by Cajal–Retzius cells. ***A***, Low-magnification overview of the hippocampal molecular layers, tested for EGFP fluorescence (top left, green), calretinin immunoreactivity (top right, red), GAD67 immunoreactivity (bottom left, blue), and of the merged signals (bottom right). ***B***, High magnification of molecular layer neurons identified as Cajal–Retzius cells by their EGFP expression (top left, green) or GABAergic interneurons by GAD67 immunoreactivity (bottom left, blue). Cajal–Retzius cells and GAD67-positive interneurons show calretinin immunoreactivity (top right, red). All signals superimposed with DAPI (white) nuclear counterstaining (bottom right). Solid arrowheads indicate Cajal–Retzius cells, outlined arrowheads indicate GAD67-positive interneurons. ***C***, left, Summary plot of the relative fraction of calretinin-positive cells that express EGFP, GAD67, or neither EGFP nor GAD67 (nnor). Right, Regional subdivision of the data in the left panel for different regions of interest. 1, CA3 subfield; 2, CA1 subfield; 3, subiculum. Notice that the vast majority of calretinin-positive cells express EGFP but not GAD67.

When we studied the immunoreactivity for the calcium-binding protein calretinin (Schwaller, 2014; Fig. 8), we found that, out of *n* = 528 labeled cells, the vast majority (*n* = 471) was EGFP-positive/GAD67-negative, with much smaller fractions being either EGFP-negative/GAD67-positive (*n* = 42) or EGFP-negative/GAD67-negative (*n* = 15). Overall, EGFP-expressing cells were in large part immunoreactive for calretinin (82.4%, *n* = 471 EGFP-positive cells). Although this result confirms previous reports of the presence of calretinin-expressing interneurons in the hippocampal molecular layers (Jiang and Swann, 1997), it also underscores the critical persistence of calretinin-positive Cajal–Retzius cells in the postnatal hippocampus, which is rarely considered. In fact, at least at the developmental stages examined here (P7–P60), Cajal–Retzius cells are, quantitatively, the largest population of cells in the vicinity of the HF. Therefore, calretinin immunoreactivity observed in a cell located in this area (especially if small in size) should be taken as putative evidence for a Cajal–Retzius cell, rather than for a GABAergic interneuron. Similarly, to what has been mentioned before for reelin immunoreactivity, we noticed the importance of high magnification. In fact, the strong signal from calretinin-expressing hilar mossy cells (Blasco-Ibáñez and Freund, 1997) may require a low acquisition gain, which may limit the detection of weaker labeling in Cajal–Retzius cells.

**Figure 9.**
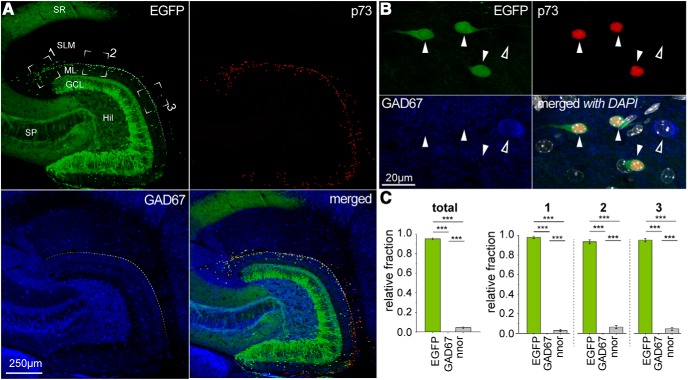
The tumor protein p73 is specifically expressed by Cajal–Retzius cells. ***A***, Low-magnification overview of the hippocampal molecular layers, tested for EGFP expression (top left, green), p73 immunoreactivity (top right, red), GAD67 immunoreactivity (bottom left, blue), and of all the merged signals (bottom right). ***B***, High magnification of molecular layer neurons identified as Cajal–Retzius cells by their EGFP expression (top left, green) or GABAergic interneurons by GAD67 immunoreactivity (bottom left, blue). Only Cajal–Retzius cells show p73 immunoreactivity (top right, red). Bottom right, All signals superimposed with DAPI (white) nuclear counterstaining. Solid arrowheads indicate Cajal–Retzius cells, outlined arrowheads indicate GAD67-positive interneurons. ***C***, left, Summary plot of the relative fraction of p73-positive cells that express EGFP, GAD67, or neither EGFP nor GAD67 (nnor). Right, Regional subdivision of the data in the left panel for different regions of interest. 1, CA3 subfield; 2, CA1 subfield; 3, subiculum. Notice that p73 was never found in GAD67-positive cells, irrespective of the region examined.

**Figure 10.**
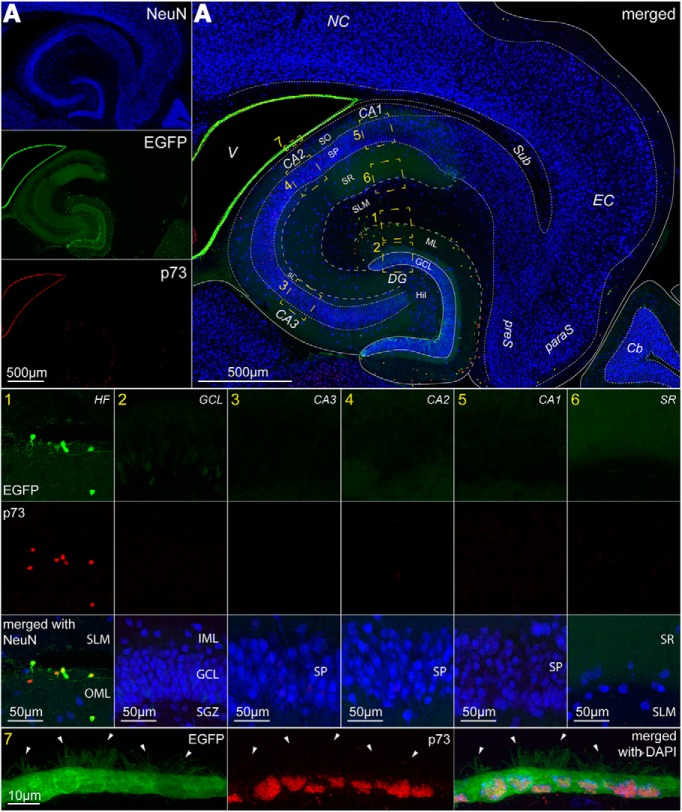
High specificity of p73 staining for hippocampal Cajal–Retzius cells in the hippocampal formation. ***A***, Low-magnification images of the hippocampus of the CXCR4-EGFP mouse with NeuN staining (top left, blue), EGFP-expression (middle left, green), and p73 immunoreactivity (bottom left, red). Please refer to the online figure to see p73 staining in this panel as it is difficult to see in the non-enlarged printed version. Right, All channels merged with boxes (1, 2, 3, 4, 5, 6, and 7) indicating regions of interest (ROIs) enlarged in the insets with labeled with the same numbers. NC, neocortex; EC, entorhinal cortex; paraS, parasubiculum; preS, presubiculum; Sub, subiculum; CA1, cornu ammonis subfield 1; CA2, cornu ammonis subfield 2; CA3, cornu ammonis subfield 3; DG, dentate gyrus; GCL, granule cell layer; ML, molecular layer; Hil, hilus; V, ventricle. Insets 1–6 show corresponding ROI at higher magnifications for EGFP expression (top), p73 immunoreactivity (middle), and signals superimposed including NeuN (bottom). Notice that p73 staining is specifically confined to EGFP-expressing cells of the molecular layers (SLM, stratum lacunosum moleculare; OML, outer molecular layer) and is absent in the other layers and regions considered. GCL, granule cell layer; IML, inner molecular layer; SP, stratum pyramidale; SR, stratum radiatum. Inset 7, EGFP positivity (left, green) and p73 labeling (middle, red) are both present in ependymal cells surrounding the ventricle (right, signals superimposed plus DAPI counterstain). Filled arrowheads indicate EGFP-positive cilia.

Lastly, we considered the cell type-specificity of p73 expression, which has been proposed to play important developmental roles in Cajal–Retzius cells and cortical patterning (Meyer et al., 2002). As shown in Figure 9, this was the only one of the four markers considered that showed very high specificity for Cajal–Retzius cells. Out of 576 p73-expressing cells, 555 were EGFP-positive/GAD67-negative, none were EGFP-negative/GAD67-positive, and a very tiny fraction of the sample (*n* = 21) was EGFP-negative/GAD67-negative. Overall, EGFP-expressing cells were consistently immunoreactive for p73 (100%, *n* = 556 EGFP-positive cells). Furthermore, p73 expression appeared specific throughout all the hippocampal areas beyond our region of interest, i.e., the molecular layers (Fig. 10, see online version for closer examination of the low-magnification panels). In addition to the HF and molecular layers (our region of interest), we examined the granule cell layer, as well as strata pyramidale and radiatum of all the CA subfields (CA3, CA2, ad CA1). In contrast to our region of interest, which showed immunopositivity for p73, these latter layers and regions did not reveal any detectable p73 labeling in NeuN-positive or NeuN-negative cells. However, immunoreactivity for p73 was surprisingly found also in ependymal cells surrounding the ventricle. Because of their localization and typical shape, this unexpected result creates, nevertheless, no interpretational ambiguities. Thus, this finding indicates that p73 is an excellent molecular marker for Cajal–Retzius cells in the postnatal hippocampus at all the developmental stages considered here (P7–P60; data not shown).

Next, we decided to test the effectiveness of these results by quantifying and comparing the developmental fractional density of neurons in the marginal zone using two of the previously studied markers: i.e., p73 and reelin. Movie 1 shows that the largest fractional density of p73-immunoreactive neurons is maintained in the hippocampus at all developmental stages examined (P7–P60). As the analysis shows, neocortical Cajal–Retzius cells in the neocortex virtually disappear with brain maturation and apoptosis (Chowdhury et al., 2010); therefore their fractional density increases in the hippocampus, where they persist. It is also interesting to note their incomplete disappearance from the periallocortex (entorhinal cortex and subicular complex). When the same analysis was performed to study reelin immunoreactivity in p73-negative neurons, results were dramatically different, with reelin-labeled cells remaining homogenously distributed throughout the entire marginal zone at all developmental stages. This is due to the presence of reelin-expressing GABAergic interneurons that are not subjected to apoptosis. We think that this result convincingly highlights the risks of using reelin as an exclusive marker for Cajal–Retzius cells.

Movie 1. Developmental profile of Cajal–Retzius cells identified by p73 immunoreactivity and reelin-positive GABAergic interneurons (p73-negative) in a model of a horizontal brain slice. Density plot (scaled to minimal/maximal density for each time point) was calculated based on measurements of P7, P14, P30, and P60 animals (*n* = 3 each, *n* = 6 per animal). Data points were calculated with linear extrapolation for P2–P7 and with linear interpolation for P7–P14, P14–P30, and P30–P60. Notice the contrast between the fading Cajal–Retzius cells (indicated by red arrowhead) and the persistent GABAergic interneurons in the neocortex (NC). 10.1523/ENEURO.0516-19.2019.video.1

**Figure 11.**
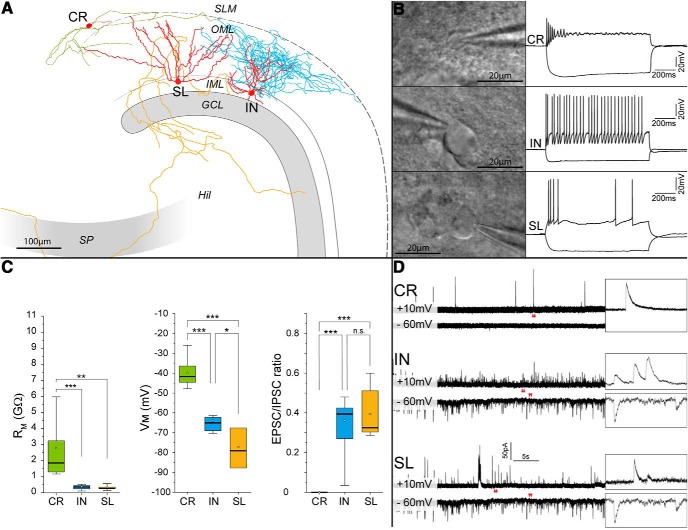
*Post hoc* anatomic reconstruction and basic electrophysiological properties of Cajal–Retzius cells, GABAergic interneurons, and semilunar cells. ***A***, Neurolucida reconstructions of biocytin-filled neurons (somato-dendritic domains in red and axons in green, Cajal–Retzius cell; blue, interneuron; and orange, semilunar granule cell), respectively. Notice the distinct morphologies of the three cell types. ***B***, top panel, Oblique contrast illumination image of a hippocampal Cajal–Retzius cell soma in an acute brain slice (left) and its respective firing pattern (right, current step: +40 pA/–60 pA, 1 s). Middle panel, Same, but for a GABAergic interneuron (current step: +280 pA/–100 pA, 1 s). Bottom panel, Dentate gyrus semilunar cell (current step: +120 pA/–100 pA, 1 s). ***C***, Summary box plots of the basic electrical properties of Cajal–Retzius cells (CR), GABAergic interneurons (IN), and semilunar cells (SL). Left panel, Membrane input resistance (R_m_). Middle panel, Membrane potential (V_m_). Right panel, EPSC/IPSC ratio. Notice the null ratio of Cajal–Retzius cells as a result of the complete absence of EPSCs. ***D***, Spontaneous synaptic currents. Top panel, Cajal–Retzius cells. The inset to the right shows the recording at higher temporal magnification of (time window marked by red arrowheads). Notice the absence of events at –60 mV. Middle and bottom panels, Same experiments, but for a GABAergic interneuron and a dentate gyrus semilunar cell, respectively. Notice the presence of synaptic events both at +10 mV (IPSCs) and at –60 mV (EPSCs).

Taken together, our data indicate that the measurement of basic morphometric parameters such as nuclear and somatic areas, coupled with immunoreactivity to a molecular marker such as p73 and/or calretinin may provide a compelling argument for the identification of hippocampal Cajal–Retzius cells in fixed tissue. However, it may be difficult to implement these criteria on individual cells previously used for electrophysiological experiments and fixed afterward. For example, withdrawing the recording electrode at the end of a whole-cell experiment may result in damage to the cell membrane and lead to the disruption of cell size and shape. Furthermore, it is not uncommon that the nucleus may remain attached to the electrode or even enter the recording pipette, which would prevent, after fixation, testing for p73 immunoreactivity. Therefore, in the following section, illustrated in Figure 11, we considered other methods easily applicable to electrophysiological experiments. Here, we have included semilunar granule cells of the dentate gyrus, which are also located in the molecular layer.

To provide a compelling anatomic identification of the cells analyzed, we filled *n* = 25 cells with biocytin. The anatomic diversity of Cajal–Retzius cells from GABAergic interneurons and semilunar granule cells can be easily recognized because of their dendritic/axonal shapes and locations. Hippocampal Cajal–Retzius cells have a stereotypical tadpole-like appearance with a single main dendrite emerging from the soma with very few, if any, secondary branches (for review, see Anstötz et al., 2018c). The location of the axon, after its emergence opposite of the main dendritic trunk, is typically restricted to the area around the fissure and molecular layers. In contrast, despite a large degree of variability because of the many subpopulations present (Freund and Buzsáki, 1996), the dendritic arborization of GABAergic interneurons is overall more complex, with several main dendritic branches and no tadpole appearance. Furthermore, their axonal cloud is usually denser and not necessarily restricted to the molecular layers (Freund and Buzsáki, 1996). The identification of semilunar cells is based on their location (outside the granule cell layer) and morphologic appearance displaying a large lateral extension of their dendritic arborization with an axon endowed with large mossy fiber boutons forming collaterals in the inner molecular layer before reaching the hilus (Williams et al., 2007).

When the electrophysiological properties of these anatomically-identified subpopulations were analyzed and compared, clear differences were observed (Fig. 11). First, the membrane input resistance of Cajal–Retzius cells (2.2 ± 0.38 GΩ, *n* = 13) was significantly larger than what found in GABAergic interneurons (0.34 ± 0.02 GΩ, *n* = 21, *p* < 0.001) or semilunar granule cells (0.32 ± 0.07, *n* = 7, *p* < 0.001). Second, the resting membrane potential measured immediately after breakthrough appeared different among all three subpopulations (*p* < 0.001), with Cajal–Retzius cells being the most depolarized (–39.49 ± 2.19 mV, *n* = 11), followed by GABAergic interneurons (–65.7 ± 1.44 mV, *n* = 6) and by semilunar granule cells, which were the most hyperpolarized (–74.38 ± 4.03 mV, *n* = 5). Lastly, in contrast to GABAergic interneurons and semilunar granule cells, which receive spontaneous inhibitory and excitatory synaptic events (EPSC frequency: 1.4 ± 0.6 Hz, IPSC frequency: 6.0 ± 2.4 Hz, ratio of their frequency: 0.32 ± 0.08, *n* = 5 and EPSC frequency: 3.9 ± 0.5 Hz, IPSC frequency: 11.0 ± 2.0 Hz, ratio of their frequency: 0.39 ± 0.05, *n* = 7, respectively), Cajal–Retzius cells lacked detectable excitatory events (EPSC frequency: 0.0 ± 0.0 Hz, IPSC frequency: 0.2 ± 0.05 Hz, ratio of their frequency: 0 ± 0, *n* = 7), as previously reported in the hippocampus (Marchionni et al., 2010) and neocortex (Kilb and Luhmann, 2001). Therefore, Cajal–Retzius cells can be functionally distinguished from other neurons located in the same layer. Thus, this approach can be useful for electrophysiological experiments (both in current-clamp and voltage-clamp) especially in the absence of a successful biocytin filling and/or anatomic recovery of the recorded cell.

Next, we examined the anatomic reconstructions obtained from our sample and generated cell type-specific dendritic and axonal density maps.

**Figure 12.**
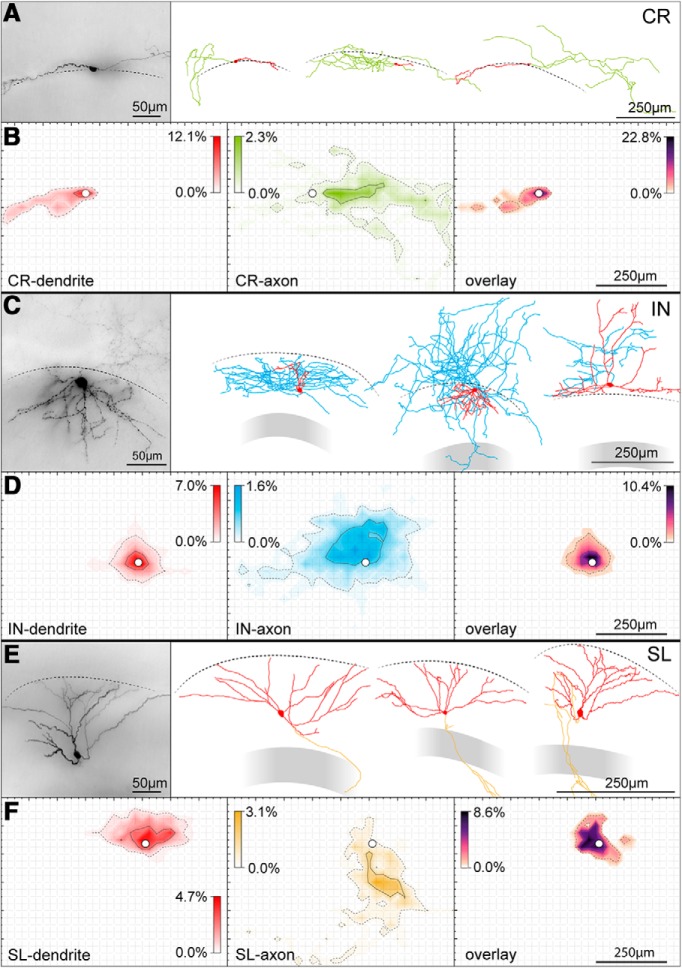
Dendritic and axonal morphology distinguish Cajal–Retzius cells from GABAergic interneurons and semilunar cells. ***A***, left panel, Micrograph of a typical Cajal–Retzius cell revealed by a DAB reaction. Dashed line represents the HF. Notice the typical bipolar morphology, with a single dendrite emerging from one pole (left) and the axon from the opposite pole (right) of the soma. Right panel: examples of three Cajal–Retzius cells, reconstructed with a Neurolucida system. Somato-dendritic domains shown in red, axons in green. ***B***, Fractional density plot of dendrites (left panel, red), axons (middle panel, green), and axo-dendritic overlay (right, high-contrast LUT). Cells (*n* = 16) were aligned at soma (white circle). Grid size is 25 × 25 µm^2^. ***C***, Same experiments as in ***A*** but for a GABAergic interneuron. Somato-dendritic domain shown in red, axons shown in blue. Notice the multipolar dendritic configuration and heterogeneity of GABAergic interneurons. The axonal arborization mainly surrounds the somato dendritic domain. ***D***, Same experiment as in ***B*** but for (*n* = 17) GABAergic interneurons. ***E***, Same experiments as in ***A*** but for a semilunar cell. Somato-dendritic domain shown in red, axons shown in blue. Notice the semilunar-like dendritic configuration directed toward the HF and stereotypical appearance of these neurons. Axonal arborizations show few segments within the molecular layers and penetrate the granule cell layer (gray) to project to CA3. ***F***, Same experiment as in ***B*** but for (*n* = 6) semilunar cells. ***G***, Sholl analysis of the dendritic domains of all the cell types (same *n* as in ***B***, ***D***, and ***F***, respectively) with a 25 µm circular increment (from soma). Lines show the means at every increment, transparent area illustrate the SD. ***H***, Box plots of the total dendritic length (left), number of dendrites emerging from cell (middle), and number dendrite endings (right). Notice the overall sparse dendritic configuration of Cajal–Retzius cells.

Figure 12 gives a quantitative description of the dendritic and axonal confidence regions as well as dendritic length and complexity for the different cell subtypes. This type of knowledge can be useful and guide the optimal placement of stimulating electrodes aimed at evoking compound postsynaptic events in the recorded cell or suggests what postsynaptic target to select in case of paired recordings. For example, in the case of Cajal–Retzius cells, an electrode placed within 250 µm from the soma in the direction of the main dendritic trunk would run the risk of a direct stimulation or damage to the cell membrane. Similarly, a location within 600 µm on the opposite side of its soma would incur the risk of antidromic stimulation of the recorded cell. In the case of experiments aimed at recording from pairs of synaptically connected Cajal–Retzius cells to interneurons, our data would suggest that interneurons in the range of the highest axonal density would be the preferential targets to be tested. Identical considerations can be done for the results of the analysis performed on interneurons and semilunar granule cells.

## Discussion

This work has begun to address misconceptions that may lead to the incorrect identification of hippocampal neurons by taking advantage of a mouse line (CXCR4-EGFP mouse) that has been repeatedly validated as an efficient tool for the identification of Cajal–Retzius cells (Marchionni et al., 2010, 2012; Cosgrove and Maccaferri, 2012; Quattrocolo and Maccaferri, 2013; Anstötz et al., 2014, 2016, 2018a,b,c, 2019). Thus, we were able to unequivocally examine the cell type-specificity of a variety of morpho-functional parameters and provide some guidelines aimed at avoiding errors in distinguishing between Cajal–Retzius cells, GABAergic interneurons and semilunar granule cells, which are all located in the molecular layers of the hippocampus. To our knowledge, this is the first quantitative study that has specifically addressed this issue and we think that our results have important practical consequences for the correct interpretation of experimental results.

### Hippocampal Cajal–Retzius cells are not GABAergic neurons

It is always difficult to reach conclusive answers when contrasting experimental results are reported in the literature. This, unfortunately, has been the case for the presence of GAD (Imamoto et al., 1994; Pesold et al., 1998), GABA (Yu et al., 2014), or lack of thereof (del Río et al., 1995; Soda et al., 2003; Stumm et al., 2003; Hevner et al., 2003; Anstötz et al., 2016) in Cajal–Retzius cells. GAD immunoreactivity would indicate the expression of the key enzyme in GABA synthesis and suggest that Cajal–Retzius cells are GABA-releasing neurons. We think that one of the major reasons leading to this ambiguity stems from the functional limits of immunohistochemical experiments. Immunohistochemistry against molecular markers used to identify Cajal–Retzius cells seldom reveals the complete and detailed morphology of the reactive cells, which is very different in Cajal–Retzius cells (stereotypical tadpole-like) compared to GABAergic interneurons (large variability, often multipolar as in neurogliaform cells; for review, see Armstrong et al., 2012). This first limitation, therefore, makes the use of immunohistochemistry alone on tissue from wild type animals potentially prone to ambiguous interpretations. Therefore, GAD immunoreactivity in cells defined by a single molecular marker as Cajal–Retzius cells (for example, reelin; Yu et al., 2014) relies completely on the specificity of the chosen marker.

Here, we have confirmed the lack of GAD67 immunoreactivity in unambiguously-identified Cajal–Retzius cells thanks to the use of the CXCR4-EGFP mouse. Furthermore, our results are strengthened by the additional evidence indicating the existence of a different spatial organization and developmental regulation of Cajal–Retzius cells versus GAD67-immunoreactive interneurons. These distinct supplemental features reinforce our interpretation that Cajal–Retzius cells are not GABAergic and support previous evidence indicating that they are, indeed, excitatory neurons (del Río et al., 1995; Soda et al., 2003; Stumm et al., 2003; Hevner et al., 2003; Ina et al., 2007; Quattrocolo and Maccaferri 2014; Anstötz et al., 2016). Our data also explain the previous misidentification of GABAergic interneurons for Cajal–Retzius cells as the consequence of interpreting results obtained using single molecular markers (Yu et al., 2014; Puighermanal et al., 2015). For example, although Cajal–Retzius cells do express reelin and are the major source of this molecule at early developmental stages (D'Arcangelo et al., 1995; Ogawa et al., 1995; Derer et al., 2001), reelin-expressing interneurons are also abundant in the hippocampal molecular layers at postnatal stages (Alcántara et al., 1998; Pesold et al., 1998a,b, 1999). Therefore, in the absence of other criteria, the co-localization of reelin and GAD should not be taken as evidence of a GABAergic phenotype for Cajal–Retzius cells. In fact, our measurements show a roughly equal fraction of reelin-expressing Cajal–Retzius and GABAergic interneurons in these regions. Similarly, we have found that the nuclear transcription factor COUP-TFII is not exclusively expressed in GABAergic interneurons (Fuentealba et al., 2010; Alzu’bi et al., 2017), but it is also present in the vast majority of Cajal–Retzius cells. Hence, the use of COUP-TFII as a molecular marker identifying specific subclasses of GABAergic interneurons (such as neurogliaform cells, Fuentealba et al., 2010; Puighermanal et al., 2015) should be preceded by ruling out the possibility that the examined neurons are Cajal–Retzius cells.

### Criteria for the identification of hippocampal Cajal–Retzius cells in morpho-functional studies

Our results suggest that a few surprisingly practical and simple criteria may allow the distinction of Cajal–Retzius cells from other neurons located in the molecular layers, even in experiments that do not reveal the complete morphology of the studied neurons.

First, Cajal–Retzius cells are generally smaller in size compared to GABAergic interneurons. This criterion can be adopted at the level of cell populations in histologic studies on fixed tissue when it is possible to measure somatic and/or nuclear diameters. This same parameter can be used as a first screening level for electrophysiological studies on living slices *in vitro*. The preselection of smaller neurons will increase the probability of targeting Cajal–Retzius cells. In contrast, if the experiment aims to obtain recordings from interneurons, smaller cells should be avoided. In addition, the direct evaluation of functional parameters such as firing pattern, membrane resting potential, and input resistance will increase the confidence in the identification of the recorded cells. Although these latter parameters may not be measured in voltage-clamp experiments performed with intracellular solutions including blockers of intrinsic conductance (such as cesium and QX-314), the lack of spontaneous synaptic events at hyperpolarized holding command voltages (close to thereversal potential of fast GABAergic transmission) appears exquisitely specific for Cajal–Retzius cells. Finally, the inclusion of intracellular labeling dyes such as biocytin in the recording whole-cell pipettes is undoubtedly the most powerful approach for the unequivocal confirmation of the neuronal identity. This, however, may not always be possible as sometimes electrode withdrawal results in loss of membrane integrity, cell death and biocytin diffusion outside the neuron of interest.

The second criterion that we propose for a reliable identification of postnatal hippocampal Cajal–Retzius cells is their expression of the p73 protein (Meyer et al., 2002; Tissir et al., 2009), which, according to our data are the only specific molecular marker for Cajal–Retzius cells when compared to reelin, COUP-TFII, and calretinin. As previously discussed, both reelin and COUP-TFII are poorly specific and can be found in interneurons of the molecular layers. Although the fraction of calretinin-expressing interneurons in the hippocampal molecular layers is low, the level of immunoreactivity of Cajal–Retzius cells appears weak and very heterogeneous, especially when compared to other hippocampal neurons (for example, mouse hilar mossy cells; Fig. 6), which may lead to experimental difficulties related to signal saturation in these latter neurons if sections are examined at low magnification.

### Implications for the design of electrophysiological experiments

Another important consequence of our study regards the design and interpretation of electrophysiological experiments. First, measurements of responses evoked by the stimulation of the hippocampal molecular layers with field electrodes should consider the persistent presence of Cajal–Retzius cells. This implies that the common interpretations of evoked events as “pure events” originated by entorhinal cortex afferents need to be taken with caution at least. In fact, optogenetic stimulation of entorhinal neurons would appear a much more selective method allowing more straightforward interpretations. Second, the density maps we have provided for Cajal–Retzius cells, GABAergic interneurons and semilunar granule cells, may form the basis for a rational selection of connected neurons in paired recording experiments and hence improve the efficiency of these (usually challenging) experiments.

## Conclusions

Our results highlight the importance of precisely recognizing the neuronal diversity of cells located in the molecular layers of the hippocampus, which perform critical, spatial-related, integrative functions. We conclude that, given the persistence in these areas of Cajal–Retzius cells of the postnatal brain, their presence needs both to be always considered in the interpretation of experimental results, as they form a non-GABAergic network that is seldom recognized. We also indicate a set of criteria for the unambiguous identification of Cajal–Retzius cells of the hippocampus, which can be easily implemented in the design of immunohistochemical and electrophysiology experiments. Lastly, we strongly caution that the use of single molecular markers believed to be specific indicators for Cajal–Retzius cells and/or interneurons should be abandoned as they may lead to ambiguous interpretations of collected data.

## Synthesis

Reviewing Editor: Menno Witter, Norwegian University of Science and Technology Decisions are customarily a result of the Reviewing Editor and the peer reviewers coming together and discussing their recommendations until a consensus is reached. When revisions are invited, a fact-based synthesis statement explaining their decision and outlining what is needed to prepare a revision will be listed below. Note: If this manuscript was transferred from JNeurosci and a decision was made to accept the manuscript without peer review, a brief statement to this effect will instead be what is listed below.

This is a transferred manuscript and the authors have appropriately responded to the comments raised by the reviewers. Both reviewers indicated that the results are important to the field by providing important tools to experimentally work on Cajal-retzius cells in the hippocampus.

I have no further comments.
