# Supplementary figures and images for: A Toolbox of Criteria for Distinguishing Cajal–Retzius Cells from Other Neuronal Types in the Postnatal Mouse Hippocampus
Source: eNeuro. 2020 Jan 22;7(1):ENEURO.0516-19.2019. doi: 10.1523/ENEURO.0516-19.2019 (PMC7004485; doi:10.1523/ENEURO.0516-19.2019)

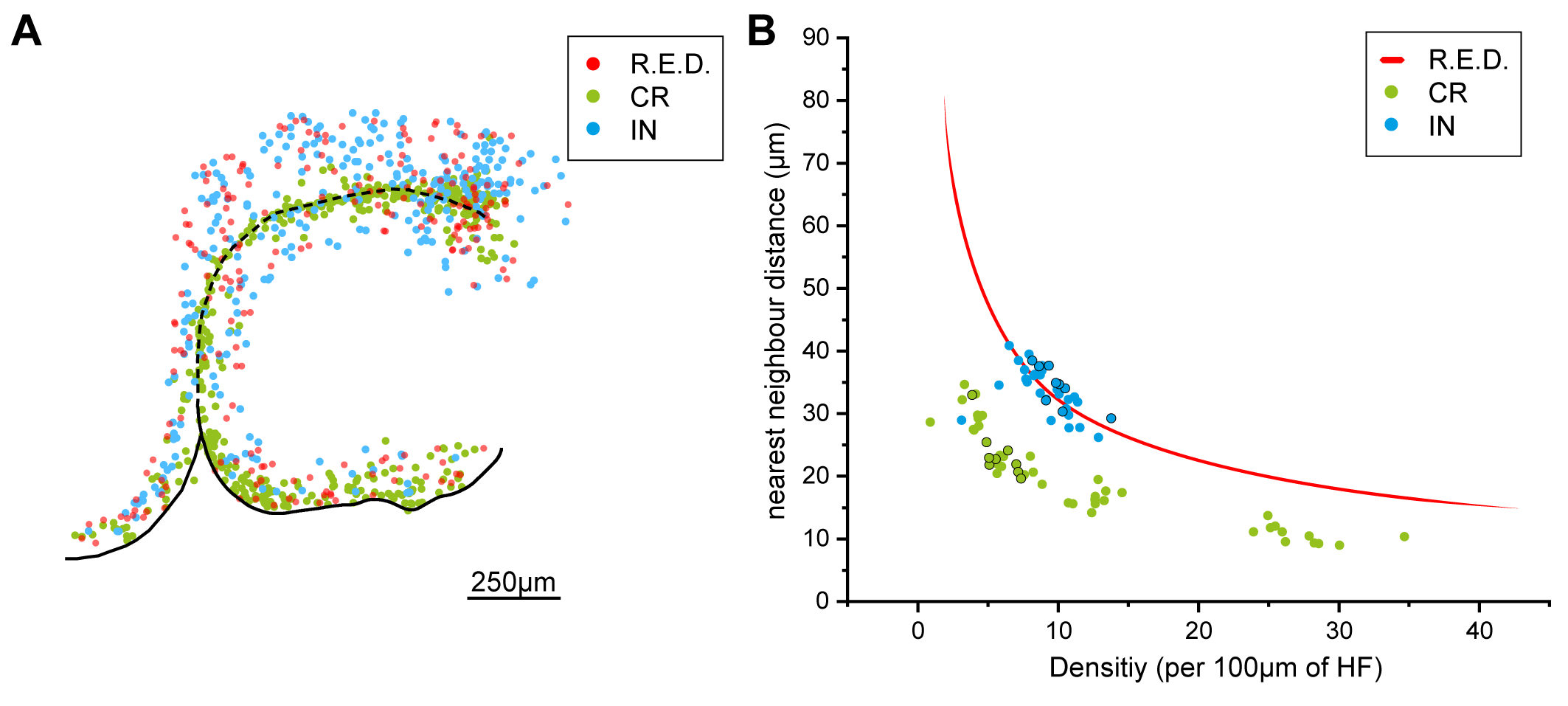

Supplement: Extended Data Figure 4-1 — Nearest neighbor distances of Cajal–Retzius cells and GABAergic interneurons compared to a simulated a random equal distribution (R.E.D.). A, Plot of Cajal–Retzius cells (n = 461), interneurons (n = 511), and R.E.D. cells (n = 500), in a normalized model of the hippocampus. B, Plot of the linear cell density of Cajal–Retzius cells (n = 45 slices) and interneurons (n = 33 slices) against their average nearest neighbor distance (per slice). The R.E.D. perfectly follows an allometric fitted function (red line, y = 111.83×−0.54; R 2 = 1), based on a simulation with n = 5× values (25 iterations each). Notice that the GABAergic interneurons are closely associated with the function of the R.E.D. suggesting their equal distribution in the hippocampal molecular layers. The distribution of Cajal–Retzius cells is shifted downwards, suggesting a clustered distribution. Selection of data points shown in A have a black outline. Download Figure 4-1, TIF file. [file sup_enu-eN-MNT-0516-19-s02.tif]
